# Prdx6 retards senescence and restores trabecular meshwork cell health by regulating reactive oxygen species

**DOI:** 10.1038/cddiscovery.2017.60

**Published:** 2017-09-11

**Authors:** Bhavana Chhunchha, Prerna Singh, W Daniel Stamer, Dhirendra P Singh

**Affiliations:** 1 Department of Ophthalmology and Visual Sciences, University of Nebraska Medical Center, Omaha,NE, USA; 2 Ophthalmology, Duke Eye Center, Duke University, Durham, NC, USA

## Abstract

A progressive decline in antioxidant potential and accumulation of reactive oxygen species (ROS) are major causes of pathogenesis of several diseases, including glaucoma. Trabecular meshwork (TM) dysfunction resulting in higher intraocular pressure (IOP) is a hallmark of glaucoma, but its causes are unclear. Using human (h) TM cells derived from glaucomatous and normal subjects of different ages and cells facing oxidative-stress, we showed that specific loss of moonlighting antioxidant protein Peroxiredoxin (Prdx) 6 in aging or in glaucomatous TM cells caused ROS accumulation and pathobiological changes in TM cells. Prdx6 limits the levels of ROS, thus preventing overstimulation of genes and resultant deleterious effects. We found that Prdx6 levels declined in aging and were reduced dramatically in glaucomatous and aged TM cells. Biochemical assays revealed enhanced levels of ROS, and high expression/activation of TGF*β*s and its responsive extracellular matrix genes *α*-SM, fibronectin, TGase2 and Tsp1 in aged or glaucomatous cells. Furthermore, hTM cells displayed typical features of the combined effects of TGF*β*s and oxidative-stress-induced cellular changes, showing increased levels of lipid peroxidation, oxidative DNA damage, and senescence markers p16, p21 and SA-*β*gal activity, along with reduced levels of telomerase expression and activity. Exposure to oxidative-stress (H_2_O_2_) or knocking down of Prdx6 (with antisense) accelerated this process. Importantly, Prdx6 delivery to sick or aged TM cells reversed the process. We propose Prdx6 as a potential therapeutic target to guard the TM from oxidative-stress and age-dependent accumulation of ROS by balancing redox-homeostasis to prevent ocular disorders, like glaucoma.

## Introduction

Several lines of evidence indicate that a progressive increase of reactive oxygen species (ROS)-evoked-oxidative-stress and loss of antioxidant molecules are major causes of age-associated degenerative disorders, including ocular diseases like glaucoma.^1–3^ Pathology in glaucoma has been linked to oxidative-stress,^4^ which can result from a decline of expression and activity of antioxidant proteins during aging.^5–9^ A common feature and risk factor of glaucoma is elevation of Intraocular pressure (IOP).^4^ The trabecular meshwork (TM) is the site that controls IOP by regulating aqueous humor (AH) outflow. Disturbance in normal functioning of the TM due to cell pathobiology caused by oxidative-stressors^10,11^ or by reduced expression and activity of antioxidant genes such as Prdx6 can lead to elevation of IOP. The TM has been reported to have high levels of metabolic products of lipid-peroxidation (LPO) and an abundance of DNA adducts.^2,3,9^ Damage in TM by oxidation and biomolecules has been proposed to be a cause of obstruction in AH outflow that may in turn results in elevated IOP.

TM cells are in continuous contact with AH, which has been shown to contain ROS that can alter the physiological functions of TM. ROS also are responsible for cellular signaling for various cytokines and growth factors including transforming growth factor *β*s (TGF*β*s).^12^ TGF*β*s have been shown to be present in ocular media in humans and other species, and their levels are increased in AH and aged/glaucomatous eyes.^13,14^ Furthermore, TGF*β*-induced overstimulation of extracellular matrix (ECM) proteins have been shown in glaucomatous/aging TM, and this process is proposed to play a part in pathobiology of TM.^12,15,16^ TGF*β*s are activated by ROS during oxidative-stress, and, notably, they are also inducers of ROS,^6,17^ acting by regulating NADPH oxidase4 (NOX4) enzymes.^18,19^ If excess ROS are not removed (with antioxidants, like Prdx6), the synergistic adverse signaling produced by TGF*β* and ROS can be deleterious to cells by overstimulating genes like ECM protein genes. Several investigations suggest that increased deposits of ECM in the TM are responsible for elevated IOP. *In vivo*, the perfusion of TM cells with H_2_O_2_ alters the mechanism of drainage of AH from anterior chamber.^20^ Furthermore, enhanced cellular oxidative load produces intraocular increased LPO in cells, and activates senescence markers such as cyclin-dependent kinase (CDK) inhibitors, p16 and p21. Induction of p16 and p21 in aging and glaucomatous TM cells is directly associated with ROS accumulation.^5^ All these findings indicate a strong relationship between oxidative-stress and senescence/aging.^21–23^ In addition, recent cell culture-based studies support the deleterious role of oxidative-stress in TM cell abnormality.^3,24,25^

Prdx6 defends many cell types through glutathione (GSH) peroxidase and calcium (Ca^++^)-independent phospholipase A_2_ (PLA_2_) activities.^6,26,27^ It has the ability to reduce phospholipid hydroperoxides, making it superior to other antioxidants. Prdx6 can protect cells from membrane, DNA, and protein damage mediated by LPO.^28,29^ A catalytic triad [Serine (S) 32, Histidine (H) 26 and Aspartic acid (D) 140] are the active sites for PLA_2_ activity, and Cysteine (C) 47 is responsible for GSH peroxidase activity.^26,30^ Prdx6 is highly expressed in cells of the TM (current manuscript), ganglia,^5^ brain, eye and lung.^6,15,26–28,31^ It is predominantly localized in the cytoplasm, but also occurs in lysosome, plasma membrane, endoplasmic reticulum, mitochondria and cerebral spinal fluid.^27,32^ In earlier studies, we showed that loss of Prdx6 makes cells highly susceptible to stressor-induced death.^6,28^ These lines of observation underscore Prdx6’s biological importance and provide rationale for the current study. On the basis of above reports and current works, we hypothesize that: (1) ROS and ROS-driven activation of TGFβs may be responsible for over-modulation of ECM genes that in turn interrupts the flow of AH, and (2) this pathogenic process can be interrupted by delivery of an antioxidant protein, Prdx6.

Herein, we test the hypothesis that aging and oxidative-stress are involved in cellular etiopathology of the TM, which is exaggerated with advancing age due to decline of the antioxidant protein Prdx6. We additionally studied whether Prdx6 deficiency in TM cells causes increased levels of intracellular ROS, and analyzed possible association with TGF*β*. Using TM cells of different ages and glaucomatous TM cells coupled with underexpression or overexpression of Prdx6 in the presence or absence of oxidants as a model system, we demonstrated that normal cellular processes were adversely affected by activation of deleterious signaling pathways by both TGF*β*s and oxidative-stress. These deleterious processes were reversed by raising Prdx6 levels, a finding which has implications for treating or delaying TM cell pathobiology and glaucoma.

## Results

### Loss of Prdx6 in aging and glaucomatous TM cells led to increased accumulation of ROS, LPO and a decline in cell growth

**Figure 1:**
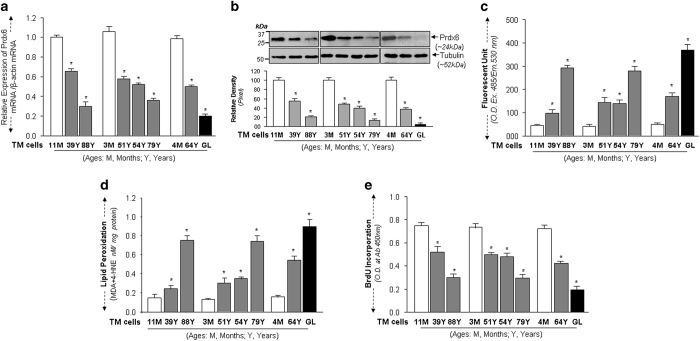
Prdx6 decline in aging triggered increased oxidative load, restricted cell growth and accumulation of LPO contents in aging and glaucomatous (GL) TM cells. (**a**, **b**) Cultured primary TM cells at passage (p) 3 to 4 derived from human subjects of variable ages were collected and processed for expression assays. (**a**) qPCR showing decline of Prdx6 transcript with age and glaucoma. Total RNA was isolated and submitted to qPCR analysis using probes specific to Prdx6 gene. (**b**) Cells were collected and cellular extracts were extracted and immunoblotted with anti-Prdx6 antibody as shown. Lower panel, histograms show densitometric analysis of protein bands of Prdx6. (**c**) Increase of ROS in normal TM cells of variable ages, and dramatic elevation in GL TM cells. Histogram values reflect mean±S.D. of three independent experiments. Asterisks indicate *P*-values from *t*-test comparisons to control (*P<0.001). (**d**) Histograms showing increased levels of LPO contents in aging TM cells and GL TM cells. Values reflect mean±S.D. of three independent experiments. Asterisks indicate *P*-values from *t*-test comparisons to control. (*P<0.001). (**e**) Age-dependent restricted cell growth in normal and GL TM cells. Cultured TM cells of variable ages and GL TM cells were subjected to BrdU assay for 24 h. Cell growth was documented in the form of histograms as indicated. Asterisk indicates significant difference (*P<0.001).

As in a previous report,^5^ we found a reduction in Prdx6 levels in glaucomatous TM cells. Expression assays with the same passages (P4) of aging TM cells revealed that aging TM cells bore an age-dependent reduction in Prdx6 expression (Figures 1a and b) that was linked to increased ROS (Figure 1c), accumulation of LPO (Figure 1d), injury associated with oxidative load, and progressive reduction in growth (Figure 1e). With advancing eye donor age (51Y onwards), ROS levels and LPO contents were dramatically increased, and the increase was inversely related to loss of Prdx6 expression (Figure 1b). These features of oxidative-stress were abundant in glaucomatous TM cells containing a very low level of Prdx6 (Figure 1, black bars). Collectively, our data argue an involvement of oxidative-stress in the aging process and glaucomatous state and is linked to a decline of Prdx6 levels before the accumulation of pathological insults to TM cells.

### Aging and glaucomatous TM cells aberrantly expressed TGF*β*s and ECM proteins

**Figure 2:**
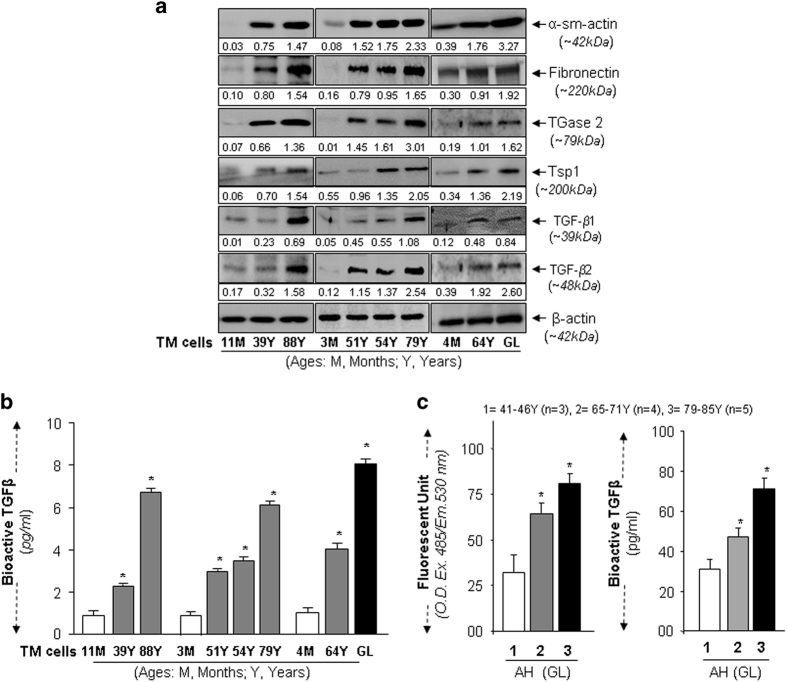
ECM protein expression increases in aging/aged and GL TM cells and was associated with higher TGF*β*s expression and activity. (**a**) Western analysis of normal and GL TM cells. Cell extracts containing equal amounts of proteins from cells of different ages and GL cells were immunoblotted using specific antibody as indicated. An age-dependent increase in ECM and TGF*β* was observed in aged and GL cells, as shown in representative immuno-stained blot. Numbers under each protein band reflect densitometry value. (**b**) A comparison of bioactive TGF*β*s in supernatants of cultured TM cells from normal subjects of variable ages, and glaucoma patients as shown. Culture supernatant were collected and bioactive TGF*β* was determined directly by the TGF*β* E_max_ ImmunoAssay system (Promega). Higher levels of TGF*β*s were detected in aged and GL TM cell supernatants. (**c**) Elevated levels of bioactive TGF*β*s in AH of glaucomatous subjects is correlated with levels of ROS. Histogram values are mean±S.D. of three independent experiments. Asterisk indicates *P*-values (*P<0.001). Increased abundance of bioactive TGF*β*s and ROS was observed.

Having determined that increased oxidative load is connected to decreased Prdx6 expression in aging and glaucomatous TM cells, we investigated the levels of ECM proteins and TGF*β*, which have been suggested as major culprits in TM pathobiology.^34^ Western analysis of younger (healthy), aged (unhealthy) and glaucomatous TM cells showed increased TGF*β*s expression in the unhealthy cells as indicated in Figure 2a. Specific examination of expression of ECM proteins (Figure 2a), such as *α*-sm-actin, fibronectin, TGase2, Tsp1 and TGF*β*s showed that their levels were significantly increased. Importantly, the increased expression was associated with loss of Prdx6 (Figure 1b). Next we examined levels of bioactive TGF*β* in TM cells.^5,6,35–37^ Assessment of bioactive TGF*β*s showed an increased pattern of bioactive TGF*β* in supernatants of aging and glaucomatous TM cells (Figure 2b), which was directly proportional to ROS accumulation (Figure 1c). This was in agreement with our previously published reports, demonstrating that increased ROS can be responsible for increased activation of TGF*β*s in aged/glaucomatous TM cells.^5,6^

### Aqueous humor derived from glaucomatous subjects showed age-dependent increase in ROS and bioactive TGF*β* levels

We next examined whether loss of Prdx6 as observed in the above experiment affect the levels of ROS and TGF*β*s in AH. Quantification established a prevalence of ROS (Figure 2c, left-panel) and bioactive TGF*β* (Figure 2c, right-panel). Notably, the levels of these molecules increased during aging and aged subjects. Nevertheless, the data show that aging indeed appeared to be connected to TM cells pathobiology, and ROS and TGF*β* may be involved, along with Prdx6, which possibly plays the major role.

### Aging led to loss of Prdx6 expression and elevated levels of TGF*β* and ECM proteins and ROS, making cells more vulnerable to oxidative-stress

**Figure 3:**
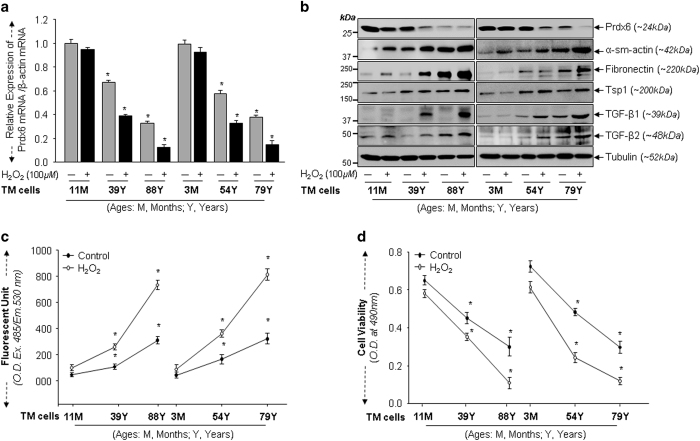
Aging TM cells exposed to H_2_O_2_ displayed reduced Prdx6 expression, increased TGF*β*s and accumulation of ECM proteins with elevated ROS levels and reduced cell viability. (**a**) Total RNA were isolated from TM cells of different ages exposed to H_2_O_2_ and processed for real-time PCR with specific primers as indicated. Histogram values are mean±S.D. of three independent experiments, showing significant reduction of Prdx6 expression in aging TM cells, in age-dependent manner (*P<0.001). (**b**) TM cells obtained from human subjects of different ages were seeded and exposed to H_2_O_2_ (100 *μ*M) and then processed for cellular extraction. Equal amounts of protein from each sample were resolved onto SDS-PAGE and immunoblotted with specific antibodies as indicated. The same membranes were probed with different antibodies followed by stripping and restripping. (**c**) Oxidative-stress induced by H_2_O_2_ increased the oxidative load more in aging TM cells than in younger ones. TM cells were exposed to H_2_O_2_, and 8 h later processed for intracellular ROS measurement by using 10 *μ*M H2-DCF-DH dye at Ex485/Em530 nm. Plotted values are mean±S.D. of three independent experiments, showing significant increase of ROS in aging TM cells (*P<0.001 compared with respective controls). (**d**) Viability assay showing that aging TM cells were more sensitive to H_2_O_2_-induced oxidative-stress, which leads to significant increase in cell death. Cells of different ages were cultured and exposed to 100 *μ*M of H_2_O_2_ as indicated. Plotted values are mean±S.D. of three independent experiments, showing significantly increased cell death in aging TM Cells (*P<0.001).

ROS generated by stressors can elevate oxidative-stress during aging.^5,6,9,29,38^ Therefore we investigated the extent to which externally applied oxidative-stress (using H_2_O_2_) affects expression levels of Prdx6, ROS, TGF*β*s and ECM proteins in aging TM cells, and TM cells vulnerability to this stress. We used TM cells of three age groups: Young, 3M and 11M (healthy TM cells); Middle, 39Y and 54Y (relatively unhealthy); and older, 79Y and 88Y (unhealthy). As predicted, a significant decline in Prdx6 expression was observed during aging, compared with untreated control (Figure 3a, gray *versus* black bars; 11M *versus* 39Y *versus* 88Y and 3M *versus* 54Y *versus* 79Y), and loss of Prdx6 was higher in aged TM cells. Next we examined how further loss of Prdx6 expression influences levels of ECM proteins and TGF*β*. Expression analysis revealed that, in cells exposed to oxidant H_2_O_2_, a decline in Prdx6 expression (Figure 3b) further upregulated expression levels of all ECM proteins tested as well as TGF*β* (Figure 3b). We then assessed functional significance of loss of Prdx6 in TM cells of variable ages after oxidative-stress. As shown in Figures 3c and d, TM cells were exposed to H_2_O_2_ to produce oxidative-stress. As expected, aging TM cells displayed elevated ROS levels and aged cells were more vulnerable to oxidation-induced death (control *versus* H_2_O_2_). Thus, loss of Prdx6 in aged TM cells appeared to cause vulnerability to oxidative damage compared with younger TM cells, suggesting an important role for Prdx6 in maintenance of TM cells.

### Loss of Prdx6 in aging or glaucomatous TM cells correlated with increased senescence markers and reduced telomerase activity

**Figure 4:**
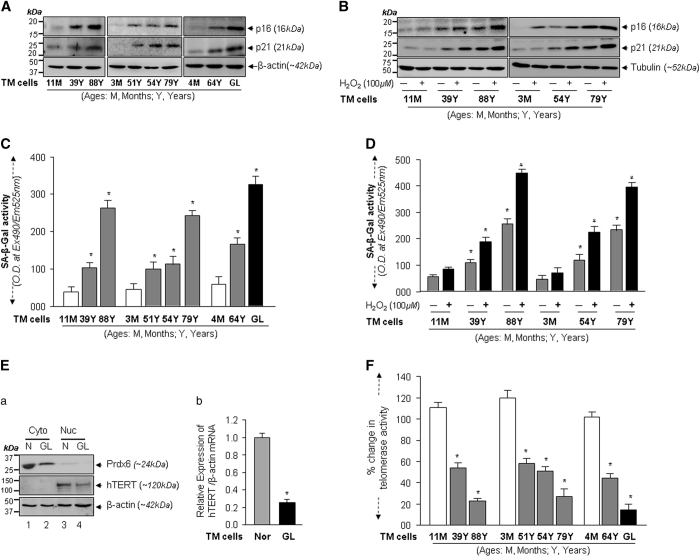
Aging/aged and GL TM cells and TM cells exposed to oxidative-stress displayed elevated cell senescence markers, p16, p21 and SA-*β*-gal activity with reduced hTERT expression and activity. (**A**) Western analysis showing increased expression of senescence markers, p16 and p21 in old normal and GL cells compared with younger normal TM cells. (**B**) Western analysis revealed that normal TM cells facing oxidative-stress showed further increases in levels of p16 and p21 in an age-dependent manner. (**C**) Lysate samples containing equal amounts of protein from normal TM cells of different ages and GL TM cells were incubated with SA-*β*-gal substrate, and SA-*β*-gal activities were measured and presented in the form of histograms. Values are mean±S.D. of three independent experiments. A significant age-dependent increase in the SA-*β*-gal activity was observed in aging/aged, and GL TM cells (*P<0.001). (**D**) Oxidative-stress increased levels of SA-*β*-gal activity in TM cells. Lysate was prepared from normal TM cells of different ages after exposure to H_2_O_2_, and was processed for SA-*β*-gal activity assay. Histogram values are mean±S.D. of three independent experiments, each with triplicate wells. An age-dependent significant increase in levels of SA-*β*-Gal activity was observed (*P<0.001), suggesting that oxidative-stress promotes cell senescence. (**E**) Expression analyses showing reduced expression of Prdx6 was related to reduced expression of hTERT (telomerase) expression in GL cells (**E**a) Cellular extracts from normal (4M old subject) and glaucomatous (56Y old subject) TM cells having equal amounts of protein were immunoblotted with Prdx6 or hTERT antibodies. *β*-actin was used as loading control. (**E**b) Total RNA was isolated from the same subjects’ TM cells, and processed for qPCR for hTERT mRNA expression by using specific probe. Histogram values are mean±S.D. of three independent experiments (*P<0.001). (**F**) Relative telomerase activity was evaluated using quantitative telomerase detection kit (QTD kit, Allied Biotech, Inc.,) in normal aging/aged and GL TM cells as indicated. Expression of telomerase activity was found to be age-dependent, and was highly reduced in GL TM cells as indicated. Histogram values represent mean±S.D. of three independent experiments (*P<0.001).

To test whether Prdx6 deficiency in aging/aged and glaucomatous TM cells overstimulates genes responsible for development of senescence, and whether oxidative-stress increases their expression, we analyzed levels of CDK inhibitors p16 and p21 and SA-*β*-gal activity. Levels of p16 and p21 protein increased in age-dependent fashion (Figure 4A), and expression levels were significantly higher in unhealthy and glaucomatous TM cells (Figure 4A; last panel; 4M and 64Y *versus* GL). Notably, levels of these molecules were increased in response to oxidative-stress (Figure 4B).

Importantly, we found that SA-*β*-gal activity significantly increased in unhealthy aging/aged TM cells (Figure 4C, open *versus* gray *versus* black bars), and staining was dramatically increased in glaucomatous TM cells (Figure 4C, 4M *versus* 64Y *versus* GL). These results revealed an age-dependent increase in SA-*β*-gal activity. Next, we examined whether oxidative-stress altered the SA-*β*-gal activity in healthy TM cells. We observed a significant increase in SA-*β*-gal activity even in cells of younger subjects (Figure 4D, gray *versus* black bars; 11M and 3M *versus* 39Y and 54Y *versus* 88Y and 79Y), suggesting that oxidative-stress triggered the onset of senescence.

Aging and oxidative-stress affects telomere shortening, as well as telomerase activity,^21,39,40^ and the antioxidant Prdx6 is known to decline with aging. Toward this end, we first examined levels of hTERT protein (Figure 4Ea) and mRNA (Figure 4Eb) expression. As expected, significantly lower expression of hTERT protein and transcript were observed in glaucomatous TM cells compared with normal TM cells (39Y), as shown in Figure 4. Furthermore, assessment of cellular telomerase activity in these cells revealed that levels of telomerase activity were dramatically reduced in glaucomatous TM cells (Figure 4F; open *versus* gray *versus* black bars). However, we did not examine levels of TERT expression in TM cells from subjects of different ages (as the expression levels can be directly related to activity as observed in Figures 4E and F; Nor *versus* GL), but we did measure the magnitude of telomerase activities in aging or aged TM cells. Quantitative telomerase activity assays showed that activity in TM cells significantly declined with aging (Figure 4F, 3M to 88Y), and was reduced significantly in aged and glaucomatous TM cells (Figure 4F; 64Y, 79Y and 88Y old).

### Antisense-mediated knockdown of Prdx6 augmented ROS levels with increased ECM proteins, LPO contents and SA-*β*-gal activity in TM cells

**Figure 5:**
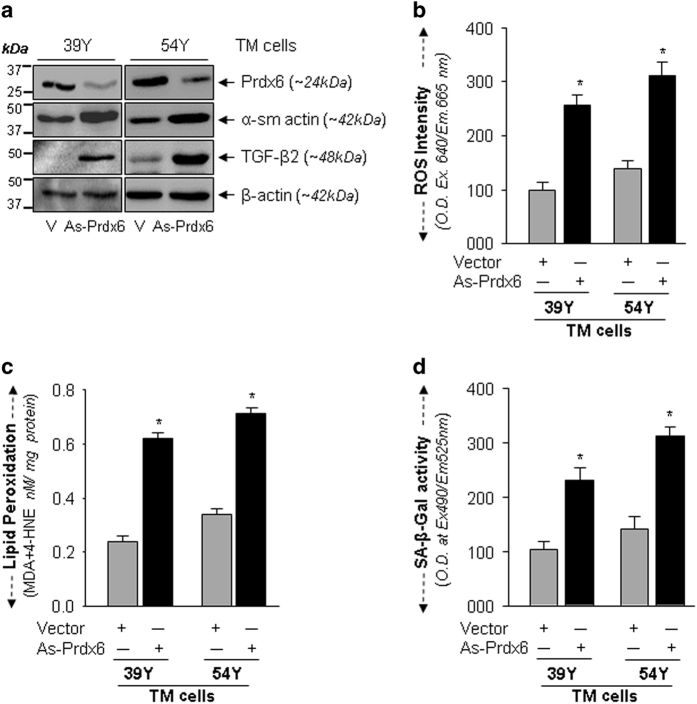
Prdx6 Knockdown experiments showing that Prdx6 deficiency contributed in the process of TM cell pathogenesis. (**a**) Normal TM cells were transfected with antisense-specific Prdx6 and enriched by selection with antibiotic. Cellular extracts from two groups (39Y and 54Y) of cells having equal amount of proteins were immunoblotted with anti-Prdx6, anti-TGF*β* and ECM protein, anti-*α*-sm-actin antibodies by stripping and restripping the same membrane. Under expression of Prdx6 in these cells enhanced the levels of TGF*β*, as well as *α*-sm-actin. (**b**–**d**) Prdx6 knockdown TM cells bore increased ROS levels with increased LPO contents and SA-*β*-Gal activity. TM cells under expressing Prdx6 were divided into three groups to assess (**b**), levels of ROS (**c**), contents of LPO levels and (**d**) levels of SA-*β*-gal activity, and were compared with empty vector transfected cells. Under expression of Prdx6 significantly increased ROS production (**b**), LPO (**c**) and SA-*β*-gal activity (**d**), suggesting that Prdx6 was indeed a cause of abnormalities in TM, and its deficiency increased pathobiological processes. Histogram values represent the mean±S.D. from three independent experiments (*P<0.001).

To investigate if Prdx6 deficiency in TM cells is indeed a cause of pathobiology, we utilized antisense specific to Prdx6 (As-Prdx6), as described previously.^5,28^ TM cells were transfected with As-Prdx6 plasmid and levels of Prdx6 protein by As-Prdx6 was verified (Figure 5a). To assess the effect of Prdx6 knockdown on TM cell biology, we examined the levels of (i) TGF*β* and *α*-sm-actin expression (Figure 5a), (ii) oxidative load (Figure 5b), (iii) LPO contents (Figure 5c) and (iv) senescence status (Figure 5d). Results revealed that Prdx6 knockdown significantly stimulated the levels of TGF*β*, ECM protein relative to transfectants with empty-vector (Figure 5). We found that Prdx6 knockdown cells had higher amounts of ROS (Figure 5b, 39Y and 54Y; gray *versus* black bars) and significantly elevated levels of LPO contents (Figure 5c, 39Y and 54Y; gray *versus* black bars), as observed in aged TM cells. Next we sought to determine whether Prdx6 deficiency could modulate the senescence process, we measured cellular senescence by SA-*β*-gal-ELISA, and found that selective knockdown of Prdx6 indeed elevated the process of senescence in TM cells (Figure 5d, 39Y and 54Y; gray *versus* black bars).

### Extrinsic expression of Prdx6 reversed abnormalities in glaucomatous TM cells and prevented the process of senescence

**Figure 6:**
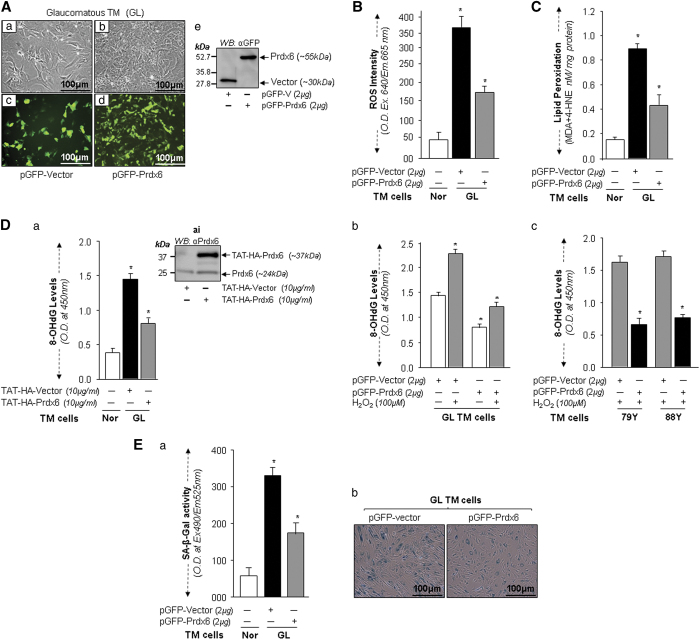
Overexpression of Prdx6 repaired GL TM cell integrity by blocking accumulation of ROS, LPO contents and oxidative DNA damage and reducing SA-*β*-gal activity. (**A**) Photomicrograph of GL TM cells expressing empty vector showing more abnormal morphology and slow growth rate (a and c) than GL-TM cells overexpressing Prdx6 (b and d). GL TM cells were transfected with GFP-vector or GFP-Prdx6 and enriched with antibiotic. (**A**e) represents Western analysis of transfectants. (**B**) Levels of ROS intensity in GL-TM cells overexpressing Prdx6 and GFP vector (Panel **B**, black bar *versus* gray bar). ROS intensity was quantified with CellRox. Histogram values are mean±S.D. of two independent experiments (*P<0.001). (**C**) Prdx6 blunted lipid peroxidation in GL TM cells. Cultured GL TM cells overexpressing Prdx6 or GFP vector for 72 h were processed for LPO assay as described elsewhere.^28^ A significantly reduced level of LPO contents was observed in GL TM cells overexpressing Prdx6 compared to GFP vector expressing ones (black bar *versus* gray bar). Histogram values are mean±S.D. of two independent experiments, (*P<0.001). (**D**a) Delivery of transduction domain (TAT)-linked Prdx6 internalized in GL-TM cells (**D**, ai), and blunted the process of oxidative DNA damage (**D**a). 8-OHdG level was measured in normal and GL TM cells pre-transduced with TAT-HA-Prdx6 or TAT-HA-Vector protein (10 *μ*g/ml) using OxiSelect TM oxidative DNA damage ELISA, as described in ‘Materials and Methods’ section. 8-OHdG levels were significantly reduced in GL TM cells supplied with Prdx6 compared with control (black bar *versus* gray bar) in 5 days. (**D**b and c) GL or aged TM cells overexpressing Prdx6 showed resistance against oxidative-stress-induced DNA damage. GL or aged TM cells overexpressing Prdx6 or its empty vector were subjected to H_2_O_2_-induced oxidative-stress and DNA oxidation was measured. Overexpression of Prdx6 offered more protection against DNA damage induced by H_2_O_2_ than did empty vector in GL (**D**b; open bars *versus* gray bars) and normal aged (**D**c; gray bars *versus* black bars) TM cells (*P<0.001). Values represent the mean±S.D. from two experiments, and are presented as histograms (*P<0.001). (**E**a and **E**b) GL TM cells overexpressing Prdx6 showed significantly diminished levels of SA-*β*-gal activity. GL TM cells overexpressing Prdx6 or vector counterpart were cultured and processed for SA-*β*-gal ELISA assay (**E**a), as well as SA-*β*-gal staining (**E**b). GL TM cells overexpressing Prdx6 displayed significantly lower SA-*β*-gal activity compared to vector transfected control (black bar *versus* gray bar), Histogram values are mean±S.D. of two independent experiments (*P<0.001). Photomicrographs are representative of SA-*β*-gal staining in GL TM cells overexpressing Prdx6 *versus* cells expressing vector only (**E**b; left panel *versus* right panel).

The experiments described above indicated that Prdx6 expression levels appear to be vital for maintaining TM cell integrity. Next, we overexpressed glaucomatous TM cells with pGFP-Prdx6 or supplied them with Prdx6 linked to transduction domain as published previously.^5,31^ The enriched transfectants with GFP-Prdx6 or GFP-vector plasmid were photomicrographed. Careful microscopic analysis revealed that TM cells overexpressing Prdx6 grew better and had improved phenotypes compared to cells containing GFP-vector only, which had more intracellular spaces, were less compact and were flattened with larger nuclei (Figure 6A). Quantification of ROS showed that Prdx6 significantly reduced ROS levels in glaucomatous TM cells (Figure 6B, black *versus* gray bar). Next, we examined the levels of LPO and oxidative DNA damage in glaucomatous TM cells overexpressing Prdx6 (Figure 6C) or transduced with transduction domain (TAT)-linked Prdx6 (Figure 6D). We found that TAT-linked Prdx6 efficiently internalized in cells (Figure 6D,ai), and was biologically active. We observed a significant reduction in LPO levels (Figure 6C, black *versus* gray bar) and oxidative DNA damage (Figure 6Da, black *versus* gray bar).

To gain further understanding of how a change in redox status would influence the level of basal oxidative DNA damage in glaucomatous and aged TM cells by addition of Prdx6, we examined levels of 8-oxoguanine (8-OH-Gua) in those cells facing H_2_O_2_-induced oxidative-stress in the presence of Prdx6. As shown in Figures 6Db and Dc, the levels of oxidative DNA damage were higher than basic levels in cells following H_2_O_2_ exposure, and were significantly higher than those in untreated cells (Figure 6Db; GL, open *versus* gray bar) and 6Dc; 79Y and 88Y, gray *versus* black bars). Interestingly, both unhealthy glaucomatous and aged TM cells overexpressing Prdx6 showed lower DNA oxidation levels, indicating that oxidative DNA damage was due to local accumulation of ROS.

Next we investigated the potency of Prdx6 in abating senescence process in glaucomatous TM cells by using SA-*β*-gal-ELISA, as well as SA-*β*-gal staining. We found that overexpression of Prdx6 significantly reduced SA-*β*-gal activity (Figure 6E, black *versus* gray bar) and SA-*β*-gal staining in these Prdx6-enriched transfectants (Figure 6Eb; GFP-vector *versus* GFP-Prdx6). By contrast, transfectants enriched with GFP plasmid showed no reduction, suggesting that Prdx6 has potential in preventing senescence-related pathobiology of cells. While Prdx6 did not completely reverse the glaucomatous TM cellular state, it at least blunted the pathobiological processes.

### Overexpression of Prdx6 in unhealthy aging/aged TM cells prevented increases in ROS levels, TGF*β* activation, LPO contents, senescence and p16 and p21 levels, and enhanced telomerase activity and proliferation

**Figure 7:**
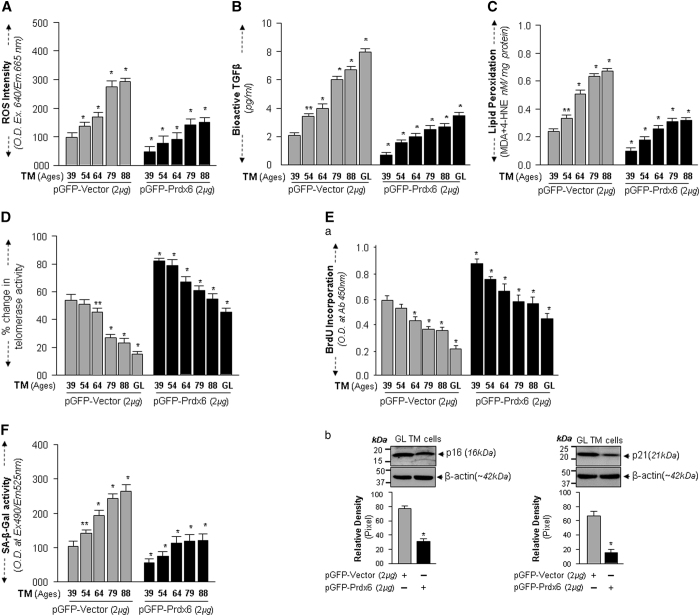
Blocking of increased ROS by means of Prdx6 delivery rescued senescence processes in aging/aged/glaucomatous TM cells. (**A**) Normal TM cells of different ages as indicated, GL TM cells overexpressing Prdx6 or TM cell transfectants with vector were seeded in 96 well plate and processed for assessing ROS levels by CellRox assay. The values (mean±S.D.) from two experiments are presented as histograms (*P<0.001). (**B**) The increased generation of bioactive TGF*β* was attenuated by Prdx6 overexpression. Transfectants expressing Prdx6 or vector were cultured, and supernatants were collected at 72 h and processed for bioactive TGF*β* assay. Values derived from two experiments are presented as histograms (mean±S.D.) as indicated. (**C**) Accumulation of LPO contents was blunted by Prdx6 overexpression. The same batch of transfectants overexpressing Prdx6 or empty vector were seeded for 72 h, and thereafter processed for LPO assay as described earlier. Histograms represent values (means±S.D.) of two independent experiments as indicated. *P<0.001, statistically significant difference. (**D**) Prdx6 overexpression increased telomerase activity in aging/aged and GL TM cells. The transfectants overexpressing Prdx6 or vector only were cultured. After 48 h transfectants were subjected to assessment of telomerase activity. The values (means±S.D.) showing telomerase activity in cells of different ages and GL cells of three independent experiments are presented as histograms. (*P<0.001). (**E**) Prdx6 overexpression released restriction on cell proliferation, and slowed CDK inhibitors. Transfectants overexpressing pGFP-Prdx6 or pGFP-Vector derived from variable ages/aged/GL TM cells were seeded and 24 h later processed for BrdU assay (**E**a) according to the manufacturer’s protocol. Histogram values are means±S.D. of three independent experiments. (*P<0.001), statistically significant difference. (**E**b) An aliquot of collected transfectants was tested for levels of p16 and p21, CDK inhibitors and markers for senescence. Cell extracts from transfectants were immunoblotted with antibodies specific to p16 and p21. The levels of p16 and p21 were reduced in GL TM cells overexpressing Prdx6 compared with the control vehicle, transfectants containing vector only. (**F**) Cell senescence assay showing increased SA-*β*-gal activity in aging TM cells. Aging TM cells overexpressing Prdx6 show reduced SA-*β*-gal activity as indicated. (*P<0.001).

Furthermore, to determine the efficiency of Prdx6 in reducing the abnormalities of aging TM cells, we utilized Prdx6 plasmid transfected TM cells. Total intracellular ROS determined by CellRox assay (Figure 7A) showed that levels of ROS were significantly lower in aging TM cells overexpressing Prdx6 than in cells expressing empty-vector (Figure 7A, gray *versus* black bars). Importantly, we also found that isolated culture supernatants from TM cells overexpression Prdx6 contained significantly reduced amounts of bioactive TGF*β* compared with empty-vector (Figure 7B, gray *versus* black bars). The result emphasizes Prdx6’s ability to normalize overstimulation of TGF*β* activity. We next examined whether Prdx6 would eliminate the increased LPO contents. Results showed that TM cells overexpressing Prdx6 attenuated LPO abundance in all aging/aged TM cells tested, compared to controls, as shown in Figure 7C (gray *versus* black bars). Furthermore, we measured telomerase activity in aging/aged and glaucomatous TM cells overexpressing Prdx6. We observed the restoration of telomerase activity in cells overexpressing Prdx6, as shown in Figure 7D (gray *versus* black bars).

Taken together, the studies provided clues that Prdx6 was able to restore abnormal cellular alterations that occur in unhealthy aged and glaucomatous TM cells. If that is the case, Prdx6 should blunt cellular senescence processes. To this end, we determined proliferation assay, levels of CDK inhibitors p16 and p21 and cellular senescence by SA-*β*-gal ELISA assay in aging and glaucomatous TM cells overexpressing Prdx6. As shown in Figure 7Ea, TM cells overexpressing Prdx6 displayed increased proliferative capability (Figure 7Ea gray *versus* black bars) and significantly decreased levels of p16 and p21 expression (Figure 7Eb, Vector *versus* Prdx6). Importantly, we observed a significantly reduced SA-*β*-gal activity (Figure 7F, gray *versus* black bars). Collectively, our results revealed that the slowing of proliferative capacity or proliferative arrest with decrease of telomerase activity and increased levels of SA-*β*-gal activity and CDK inhibitors in aging and glaucomatous cells, can be halted or blunted by Prdx6, suggesting that Prdx6 may have therapeutic potential to block oxidative-stress-associated pathological signaling.

## Discussion

Prdx6, has exceptional cytoprotective qualities and protects many types of cells.^7,26–28,31,41^ Herein, we explored the role of Prdx6 in TM cell pathobiology, specifically how its expression levels affects TM cell health during normal physiological conditions, as well as during oxidative-stress. Our study revealed that loss of Prdx6 in TM cells has several negative effects: (a) has a major impact on redox-state of unhealthy aged/glaucomatous TM cells, (b) permits accumulation of ROS, (c) increases LPO and oxidative DNA adducts, (d) arrests proliferation and enhances CDK inhibitors, p16 and p21, (e) overstimulates TGF*β* and ECM proteins, and (f) accelerates the process of senescence. These phenomena are promoted by externally applied oxidative-stressors (H_2_O_2_, in current work), and, importantly, delivery of Prdx6 in these cells reverses the processes.

On the basis of these results, we propose, for the first time, that loss of Prdx6 can be associated with TM pathobiology resulting from age-related oxidative-stress. In studying how and to what extent the relative decline of Prdx6 during aging and its dramatic loss in aged and glaucomatous TM cells influences etiopathological alteration, we observed that increased ROS-dependent injuries increased with age; the increase was dramatically higher in unhealthy aged and glaucomatous TM cells (Figures 1 and 2). At normal physiological conditions, oxidative-stress usually results from excessive ROS production due to impaired antioxidants, which in turn damages proteins, lipids, mitochondria, and DNA and leads to further impairment of cellular integrity and functionality. Oxidative-stress can be generated at elevated rates during normal aging, as well as in acute or chronic pathophysiological conditions.^42^ We found similar patterns of increased ROS with aging and ROS-dependent intracellular accumulation of oxidative contents. High levels of ROS and lower antioxidant activity have been reported in aging cells of many types and are associated with LPO, DNA damage and protein oxidation.^43^

Elevated IOP due to TM cells abnormalities leads to an increase in resistance to AH outflow at the TM. The hTM is highly sensitive to oxidative damage.^44^ Recent evidence shows that oxidative-stress plays a pivotal part in the loss of physiological functions. TM cells are in direct contact with H_2_O_2_ and TGF*β*s in AH. TGF*β*s have been measured at high levels in the AH of aged/glaucomatous eyes.^45–47^ Our study shows that TGF*β* activity is increased with age and is high in aged/glaucomatous TM cells (Figure 2) and is associated with reduced expression of Prdx6 (Figure 1). We also observed that levels of ROS and bioactive TGF*β* were profoundly increased in AH derived from glaucomatous subjects as shown in Figure 2c. ROS are activators of TGF*β*; we believe that secreted TGF*β* is activated by ROS prevalent in AH. In addition, eyes are normally exposed to sunlight (UV), which induces ROS and further accelerates the deleterious process within AH and neighboring environment. Furthermore, activated TGF*β*s generate ROS through activation of NOX4, and in turn higher ROS and TGF*β* (produced via vicious feed-forward process within cellular microenvironment) act adversely on TM cell biology. Our earlier and present experiments demonstrate that ROS-driven TGF*β* activation and TGF*β*-induced overstimulation of ECM genes/proteins might interrupt outflow of AH. Thus, our study provides a clue that under normal physiological conditions, aging itself is a cause of loss of Prdx6, leading to increased ROS accumulation-mediated TM’s abnormalities.

Deficiency of cellular antioxidants resulting from aging processes can lead to enhanced accumulation of ROS. Our results confirmed this notion, as we found that with aging there was increased ROS and ROS-related pathobiology in TM cells. We also found that aged or glaucomatous cells faced with H_2_O_2_-induced oxidative-stress showed increased ROS-induced accumulation of LPO, oxidative DNA, ECM proteins and TGF*β*. Also these unhealthy aged cells were more susceptible to oxidative death (Figure 3). In addition, we observed that aged cells displayed significantly increased expression of CDK inhibitors, p16 and p21, and SA-*β*-gal activities along with further reduction in Prdx6 expression. We conclude that both internal and external oxidative-stresses contribute to TM cell pathobiology during aging, and external stress accelerates the pathological process (Figure 4). Nonetheless, we did find that the aging process is involved in TM cell pathobiology. Furthermore, increased oxidative-stress has been reported to cause accumulation of senescent cells in tissue.^48^ We found that aging itself is a cause of senescence; biochemical analysis of aged and glaucomatous TM cells showed increased senescence markers, p16, p21 with increased SA-*β*-gal activity, and oxidative-stress accelerated SA-*β*-gal activity (Figure 4D), which was accompanied by reduction in telomerase activity with reduced Prdx6 expression (Figures 4E and F). The activity and expression were similar to that of unhealthy aging TM cells, suggesting that glaucomatous cells followed the same path as aged cells. Telomerase activity has been reported to decrease with age in normal organisms.^49^ Collectively, the above findings suggest that oxidative-stress and aging are both responsible for TM cell senescence.

To better understand whether loss of Prdx6 with aging has a direct impact on development of oxidative pathobiology, our experiment with specific knockdown of Prdx6 in TM cells revealed that loss of Prdx6 in aged/glaucomatous TM cells and cells with knocked down Prdx6 had augmentation of not only ROS levels, but also basal levels of expression and activation of TGF*β* and its related ECM genes including LPO and senescence activity (Figures 1, 2, 3, 4, 5). This suggests that ROS accumulation contributes highly to the onset of pathological abnormalities in TM. Importantly, the current study showed that sick/aging cells overexpressing Prdx6 showed reversal of TM cell phenotype (Figure 6A), and significant reduction in levels of ROS, LPO and oxidative DNA contents (Figures 6B–D), as well as senescence markers and SA-*β*-gal activity (Figure 6E). Furthermore, as shown in Figure 7 we observed that Prdx6, when overexpressed, could release arrested cell proliferation as well as break the process of senescence. Moreover, also we observed that Prdx6 rescued aged or glaucomatous cells, which are highly vulnerable to oxidative death, from externally applied oxidative-stress, H_2_O_2_. On the basis of our results we questioned what underlying factor determines whether cells adopt the senescence pathway rather than cell death or apoptosis. We reasoned this may be related to levels of ROS that affect the magnitude of DNA oxidation; greater oxidative DNA damage leads to apoptosis, while lower but elevated levels of intracellular ROS cause less DNA damage, leading to cell senescence, as evidenced by the vulnerability of aged and glaucomatous TM cells to oxidative-stress (Figure 7).^5^

However, our study reinforces the potential role of oxidative-stress in the appearance of various pathological processes. Prdx6 expression may be essential to reverse adverse pathological processes and maintain TM cell integrity and function. Further detailed studies will be needed to reveal the role of Prdx6 expression and its contribution in TM cell biology and its correlation to elevation of IOP, aging and oxidative-stress. However, based on our proof-of-principle experiments, we propose that loss of Prdx6 can be a plausible cause of etiology and progression of TM pathobiology, while introduction of Prdx6 has potential as a therapeutic intervention to blunt TM cell pathobiology-and/or prevent the process of senescence that occurs with aging and oxidative-stress. That, in turn, may ameliorate increased IOP-mediated glaucoma.

## Materials and methods

### Cell culture

Primary human Trabecular Meshwork (hTM) cells from normal (3M, 4M, 11M, 39Y, 51Y, 54Y, 64Y, 79Y and 88Y) and glaucomatous (56Y) subjects were isolated and characterized and cultured as described previously.^33^ hTM cells were maintained in Opti-MEM and/or Dulbecco’s modified Eagle’s medium (DMEM; Invitrogen, Carlsbad, CA, USA) with 0.2% BSA or 1 or 10% fetal bovine serum (FBS; Atlanta Biologicals, Inc., Flowery Branch, GA, USA) and antibiotic solution containing 100 *μ*g/ml streptomycin, 100 U/ml penicillin and 292 *μ*g/ml L-glutamine in 5% CO_2_ environment at 37 °C, as described previously.^5^ hTM cells were cultured in 96, 24, or 6 well plates and petri dishes according to the requirements of the experiment(s). TM cells reaching 80–90 percent in culture were used for the experiments. All the experiment were performed between passages (P) 3–6. The same passage and batch of TM cells were used for the experimentation to avoid any effects of passages on modulation of molecules expression to be examined. In aged hTM cells growth is halted, and they undergo the senescence process, unlike younger hTM cells. Therefore, in the following text, aged cells will be designated as unhealthy TM cells, and younger cells as healthy.

### Western blot analysis and antibodies

Total lysates of TM cells were prepared in ice-cold radio immunoprecipitation assay (RIPA) lysis buffer, as described previously.^6^ Equal amounts of protein samples were loaded onto 7.5%, 10%, 12% or 4–20% SDS PAGE gel, immunoblotted onto PVDF membrane (Perkin Elmer, Waltham, MA, USA) and immunostained with primary antibodies at appropriate dilutions. The following antibodies were used: Prdx6 monoclonal (Lab Frontier, Seoul, Korea), p16 (sc-467; Santa Cruz Biotechnology, Inc., Dallas, TX, USA), p21 (sc-397; Santa Cruz Biotechnology, Inc.), Transglutaminase2 (TGase2, sc-48387; Santa Cruz Biotechnology, Inc.), TGF-*β*1 (sc-146; Santa Cruz Biotechnology, Inc.), TGF-*β*2 (sc-90; Santa Cruz Biotechnology, Inc.), Thrombospondin1 (Tsp1, sc-59887; Santa Cruz Biotechnology, Inc.), *α*- smooth muscle-actin (*α*-sm-actin, ab5694; Abcam), Fibronectin (F3648: Sigma-Aldrich, Saint Louis, MO, USA). Membranes were incubate with horseradish peroxidase-conjugated secondary antibodies (anti-mouse, sc-2055 and anti-rabbit, sc-2054; Santa Cruz Biotechnology, Inc.). Specific protein bands were visualized by incubating the membrane with luminol reagent (sc-2048; Santa Cruz Biotechnology, Inc.) and the images were recorded with a Fujifilm-LAS-4000 luminescent image analyzer (Fujifilm Medical Systems Inc., Hanover Park, IL, USA).To ascertain comparative expression and equal loading of the protein samples, the membrane stained earlier was stripped (also restriped if needed) and re-probed with tubulin (Abcam, Cambridge, MA, USA) or *β*-actin (Sigma-Aldrich) antibody or other antibodies shown.

### Quantitative real-time PCR analysis

Total RNA was isolated using the single-step guanidine thiocyanate/phenol/chloroform extraction method (TRIzol reagent; Invitrogen) and converted to cDNA using Superscript II RNAase H-Reverse Transcriptase. Quantitative real-time PCR (qPCR) was performed with SYBR Green Master Mix (Roche Diagnostic, Indianapolis, IN) in a Roche LC480 Sequence detector system (Roche Diagnostic). PCR conditions consisted of 10-min hot start at 95 °C, followed by 45 cycles of 10 s at 95 °C, 30 s at 60 °C, and 10 s at 72 °C. Primer sequence was as follows: Prdx6, Forward: 5′- GCATCCGTTTCCACGACT-3′; Reverse: 5′- TGCACACTGGGGTAAAGTCC-3′; hTERT, Forward: 5′**-** GACGTCTTCCTACGCTTCATG-3′; Reverse: 5′- GGCATCTGAACA AAAGCCGTG-3′ and *β*-actin, Forward: 5′- CCAACCGCGAGAAGATGA-3′; Reverse: 5′- CCAGAGGCGTACAGGGATAG-3′. Expression levels of target genes were normalized to the levels of *β*-actin as an endogenous control in each group. The comparative Cp method was used to calculate relative fold expression levels using the Light Cycler 480 software release 1.5.0SP3.

### Quantitation of intracellular ROS level by H2-DCF-DA and CellROX Deep red reagent

Intracellular ROS level was measured using fluorescent dye dichlorofluorescein diacetate (H2-DCF-DA), a nonpolar compound that is converted into a polar derivative (dichlorofluorescein) by cellular esterase after incorporation into cells. DCF fluorescence is not specific for H_2_O_2,_ and other reactive species like O_2_^−^, NO and so on are also able to oxidize H2-DCF-DA into DCF. Thus, DCF fluorescence reflects overall oxidative-stress. However, on the day of the experiment, the medium was replaced with Hank’s solution containing 10 *μ*M H2-DCF-DA dye, and cells were incubated. After 30 min levels of ROS (intracellular fluorescence) were detected at excitation (Ex) 485 nm and emission (Em) 530 nm by Spectra Max Gemini EM (Molecular Devices, Sunnyvale, CA, USA).

ROS levels were measured according to the company’s protocol (CellROX Deep Red Oxidative Stress Reagent, Catalog No. C10422). In brief, TM (5×10^3^) cells derived from different ages of normal as well as glaucomatous subjects were transfected with pGFP-Vector and pGFP-Prdx6 and were enriched by multiple transfection. Transfectants were seeded in 96 well plate, and 48 h later CellROX deep red reagent was added with final concentration of 5 *μ*M, and cells were incubated at 37 °C for 30 min. Media containing CellROX deep red reagent were removed and fixed with 3.7% formaldehyde. After 15 min fluorescence signals were measured at Ex640 nm/Em 665 nm with Spectra Max Gemini EM (Molecular Devices).

### Cell viability assay

A colorimetric MTS assay (Promega, Madison, WI, USA) was performed as described earlier.^6^ This assay of cellular proliferation/viability uses 3-(4, 5-dimethylthiazol-2-yl)-5-(3-carboxymethoxyphenyl)-2 to 4-sulphophenyl) 2H-tetrazolium salt. When added to medium containing viable cells, MTS is reduced to a water-soluble formazan salt. The A490 nm value was measured after 2 h with an ELISA reader. Results were normalized with absorbance of the untreated control(s).

### Lipid Peroxidation assay

Lipid Peroxidation assay (LPO) assay was carried out according to the manufacturer’s protocol (Lipid Peroxidation Microplate Assay Kit; Oxford Biomedical Research, MI, USA) and our published report.^28^ This assay is based on the reaction of a chromogenic reagent, N-methyl-2-phenylindole (R1), with malondialdehyde (MDA) and 4-hydroxyalkenals at 45 °C. One molecule of either MDA or 4-hydroxyalkenal reacts with two molecules of reagent R1 to yield a stable chromophore with maximal absorbance at 586 nm. Briefly, normal aged/aging and glaucomatous TM cells (2×10^5^) non-transfected normal or transfected with pGFP-Vector, pGFP-Prdx6 and antisense specific to Prdx6 (As-Prdx6) were seeded in 35 mm plates, and after 72 h Cells washed twice with ice-cold PBS, and the total cell lysates were prepared as described previously.^6,28^ Equal amounts of protein were used for the assay. Optical density (O.D.) measured at 586 nm with Spectra Max Gemini EM (Molecular Devices).

### Senescence-associated (SA)-*β*- Galactosidase assay

#### (i) SA-*β*-gal Activity by ELISA

*β*-Galactosidase Detection Kit (Fluorometric) (ab176721, Abcam) was used to measure the activity following the company’s protocol. Briefly, cell lysate prepared from normal cells of variable ages, glaucomatous TM or TM cells overexpressed with pGFP-Vector and pGFP-Prdx6 or under-expressed with antisense specific to Prdx6 (As-Prdx6) with or without H_2_O_2_ treatment. 50 *μ*l standard and unknown sample (diluted in 1× lysis buffer) was added to 96 well plate followed by 50 *μ*l of FDG working solution to each well, and then plates were incubated at 37 °C for 4 h. Quantified *β*-galactosidase activity was recorded by measuring the fluorescence intensity through microplate reader at Ex490/Em525 nm after addition of 50 *μ*l stop solution with Spectra Max Gemini EM (Molecular Devices).

#### (ii) Senescence-associated (SA)-*β*-gal assay

We used Senescence *β*-Galactosidase Staining kit (9860S; Cell Signaling Technology, Inc., Danvers, MA, USA) to detect *β*-galactosidase activity at pH 6 in accordance with the company’s manual. Briefly, cells were cultured and cell monolayers were washed twice with PBS and then fixed with fixative solution (Paraformaldehyde) for 15 min. The cells were then washed twice with PBS. Staining solution (930 *μ*l Staining Solution; 10 *μ*l Staining Supplement A; 10 *μ*l Staining Supplement B; 50 *μ*l 20 mg/ml X-gal in DMF) was applied and then the cells were incubated at 37 °C for 16 h. After incubation, the cells were washed twice with PBS and photomicrographed using Camera attached NikonECLIPS Ti microscope.

### TAT-HA-Prdx6 recombinant protein purification

A full-length cDNA of Prdx6 from a human LEC cDNA library using Prdx6-specific Forward (5′- GTCGCCATGGCCGGAGGTCTGCTTC-3′ contained *NcoI* site) Reverse (5′- AATTGGCAGCTGACATCCTCTGGCTC-3′) was ligated into a TA-cloning vector (Invitrogen), plasmid consisting cDNA was amplified cloned into a pTAT-HA expression vector at *NcoI* and *Ec*oRI sites (a kind gift of Dr. SFD). Recombinant proteins were purified from transformants (*Escherichia coli* BL21 (DE3)) using QIAexpress Ni-NTA Fast Start kit column (Qiagen Inc., Valencia, CA, USA) as described.^29^ This purified protein can be either used directly or aliquotted and stored frozen in 10% glycerol at −80 °C for further use.

### 8-hydroxydeoxyguanosine estimation

The formation of 8-hydroxydeoxyguanosine (8-OHdG) is a byproduct of ROS-mediated DNA damage with oxidative-stress. The Oxiselect TM oxidative DNA damage ELISA kit is used for the quantitative measurement of 8-OHdG. In brief, genomic DNA isolated from aging or aged normal or glaucomatous TM cells or transfected with pGFP-Vector and pGFP-Prdx6 and/or transduced with TAT-HA-Vector and TAT-HA-Prdx6 with or without H_2_O_2_ exposure, and converted to single stranded DNA. DNA samples were digested with nuclease P1 (Catalog No. N8630, Sigma) followed by incubation with 10 units of alkaline phosphate (Catalog No. P5931, Sigma) for 1 h at 37 °C in 100 mM Tris, pH 7.5. 50 *μ*l of 8-OHdG standard or samples was added to the well of the 8-OHdG conjugate coated plate and incubated for 10 min at room temperature (RT). Thereafter, 50 *μ*l diluted anti-8-OHdG antibody was added to each well and incubated for 1 h at RT. The wells were washed with washing buffer and 100 *μ*l of diluted secondary antibody-enzyme conjugates were added to all wells, which were then incubated for 1 h at RT. After washes, the peroxidase activity was detected by the addition of 100 *μ*l of substrate solution and incubated for 5 min. The reaction was stopped with 100 *μ*l of stop solution and readings were taken at 450 nm with spectrophotometer (DTX 880 Multimode Detector, Molecular Device). Data were standardized to total DNA concentration and recorded.

### Detection of bioactive TGF-*β* activity

Bioactive TGF*β* in aqueous humor from glaucomatous subjects of variable ages as well as in culture supernatant from aging and glaucomatous TM cells was examined directly by using TGF-*β*1 E_max_ ImmunoAssay System (Promega). Briefly, 96-well plates were coated with TGF-*β* Coat mAb in carbonate coating buffer overnight at 4 °C, which bound soluble TGF-*β1* from solution. Standard and equal volumes of each sample were added to each well after blocking and washing the wells and were incubated for 2 h at room temperature. Captured TGF-*β* was bound by a polyclonal antibody specific for TGF-*β*. After washing, the amount of specifically bound polyclonal antibody was measured using a specific antibody conjugated to horseradish peroxidase. Absorbance of samples was read at 450 nm using a plate reader (DTX 880 Multimode Detector, Molecular Device).

### BrdU incorporation assay

Cell proliferation assay was performed with BrdU incorporation assay kit according to manufacturer’s protocol (Roche Diagnostics). Briefly, normal and glaucomatous TM cells were transfected with pEGFP-Vector, pGFP-Prdx6 were seeded into 96-well plates at a density of 5×10^3^ cells per well. Following incubation cells were labeled with BrdU for 24 h and O.D. was measured at 450 nm (DTX 880 Multimode Detector, Molecular Device).

### Construction of Prdx6 antisense

A human lens epithelial cell cDNA library was used to isolate Prdx6 cDNA having a full-length open reading frame. A full-length Prdx6 antisense (Prdx6-As) construct was made by sub cloning Prdx6 cDNA into a pcDNA3.1/NT-GFP-TOPO vector in reverse orientation. Plasmid was amplified following TOP 10 bacterial cells transformation as described earlier.^6,28^

### Telomerase (Human Telomerase reverse transcriptase (hTERT)) activity assay

Telomerase activity was evaluated using quantitative telomerase detection kit (QTD kit, Allied Biotech, Inc., Vallejo, CA, USA). Briefly, cells were lysed in 1× lysis buffer and incubated at 4 °C for 30 min. The lysate was then centrifuged at 12 000 *g* for 20 min at 4 °C, and the supernatant was collected. The protein concentration of the cell lysate was determined using a BCA protein assay. Standards, inactivated samples, and template-free reactions were also assayed on every plate for quality control. Each sample was analyzed in triplicate. Real-time amplifications were performed on Roche Quantitative PCR machine. A comparative threshold cycle (C_T_) was used to determine telomerase activity, which is negatively related to the C_T_ of real-time PCR.

### Statistical method

Data are presented as mean±S.D. of the indicated number of experiments. Data were analyzed by Student’s *t*-test. A *P* value of **P<0.050 and *P<0.001 was defined as indicating a statistically significant difference.

## Additional information

**Publisher’s note:** Springer Nature remains neutral with regard to jurisdictional claims in published maps and institutional affiliations.
